# Two new species of kidney fluke (Trematoda: Renicolidae) from New Zealand penguins (Spheniscidae), with a description of *Renicola websterae* **n. sp.**

**DOI:** 10.1007/s11230-025-10219-x

**Published:** 2025-03-01

**Authors:** B. Presswell, J. Bennett

**Affiliations:** Department of Zoology, University of Otago, P.O. Box 56, Dunedin, New Zealand

## Abstract

This study describes *Renicola websterae* **n. sp.**, a newly identified kidney fluke (Renicolidae: Trematoda) infecting two penguin species from New Zealand, the little blue penguin (*Eudyptula novaehollandiae*) and the Fiordland crested penguin (*Eudyptes pachyrhynchus*). Morphological and molecular analyses, including phylogenies based on *cox1* and 28S genes, confirmed the distinctiveness of *R*. *websterae*. Key morphological features were discerned to be statistically comparable across five developmental stages, facilitating detailed characterization even in less mature specimens. A putative second, genetically distinct *Renicola* sp. was identified in Fiordland crested penguins and intermediate fish hosts, indicating a potential trophic link, and partly completing the known life cycle. We discuss the fact that kidney flukes have only been found in these two penguins and not in other New Zealand species, and the ecological and host-specificity factors likely influencing parasite distribution. This work represents the first record of a named *Renicola* species from New Zealand and only the second species found in penguins worldwide.

## Introduction

Trematodes of the family Renicolidae are found in the kidney tubules and ureters of aquatic birds that prey on molluscs and fishes (Gibson, 2008). Two genera are currently recognised within the family; *Renicola* Cohn, 1904 and *Nephromonorcha* Leonov, 1958 (Gibson, 2008). The higher classification of the family has been a matter of some debate (reviewed in Gibson, 2008), but phylogenetic analyses now firmly place the Renicolidae within the superfamily Microphalloidea (Olson et al., 2003). Notwithstanding a number of published identification keys (Dollfus, 1946; Wright, 1954, 1956, 1957; La Rue, 1957; Odening, 1962; Riley & Owen, 1972; Sudarikov & Stenko, 1984; Gibson, 2008), the status of many species remains uncertain. This uncertainty arises from the challenges in observing critical features, primarily due to the overwhelming number of eggs present in the uterus of mature specimens.

New Zealand’s penguin species hold significant cultural and ecological importance. The country hosts the world’s largest diversity of penguins, being home to seven of the 19 extant species. Five of these species are endemic to New Zealand and its islands, and all but one are vulnerable or endangered, most with declining populations (Robertson et al., 2021). Our knowledge of the parasite fauna of New Zealand penguins remains little known except for the little blue penguin *Eudyptula novaehollandiae* (Stephens), whose parasite communities were documented by Bennett et al. (2021). The endemic Fiordland crested penguin *Eudyptes pachyrhynchus* Gray, or tāwaki, primarily inhabits the southwestern coast of the South Island, particularly within Fiordland National Park, but vagrants occasionally appear on shores all around New Zealand. Little blue penguins, known as kororā in New Zealand, are the world’s smallest penguins, and are found on the coasts of southern Australia and New Zealand (HBW & BirdLife International, 2024). Recent morphometric and genetic studies have proposed the existence of two main species, *Eudyptula novaehollandiae* from the coasts of South Australia and coastal Otago (New Zealand), and *Eudyptula minor* (Forster) endemic to the rest of New Zealand (Grosser et al., 2015, 2017). All little blue penguins in this study were collected from around the Otago coast; therefore, we have assigned these specimens to *E. novaehollandiae* (Otago little blue penguin), as opposed to *E. minor* (New Zealand little blue penguin) (see Grosser et al., 2015, 2017).

During an ongoing parasitic study of birds in New Zealand, a number of little blue and Fiordland crested penguins were discovered to harbour trematodes in their kidneys. DNA sequence and morphological comparisons with other species in the genus *Renicola* confirmed that these specimens were new to science. This study presents a description of the new species using both morphological and molecular techniques, along with phylogenetic analyses based on *cox*1 and 28S DNA genes, and aims to place the species within the context of the genus. Additionally, a potential second, genetically identified, species from a Fiordland crested penguin is briefly described and included in the phylogenies. We propose a method for artificially recognising five developmental stages of the adult worm, which allows for the morphological description of sub-mature specimens without compromising the accuracy of measurements compared to fully mature adults.

This study represents the first report of a named *Renicola* species in New Zealand, and the second species to be recorded, worldwide, from a penguin host.

## Materials and methods

### Bird collection and trematode sampling

The kidneys from a total of 10 Fiordland crested penguins and 25 Otago little blue penguins were examined for helminths between 2020 and 2024. Birds were donated after death or euthanasia by the Dunedin Wildlife Hospital or Department of Conservation, and were frozen immediately post mortem. Birds were defrosted, the kidneys removed, dissected and examined under a dissecting microscope. Kidney parasites were preserved in 70% ethanol for whole-mount, and 96% ethanol for genetic analyses, or formalin for SEM photography.

### Morphological data

Trematodes were stained using iron acetocarmine, dehydrated through a graded ethanol series, cleared in clove oil and mounted in Canada balsam. Measurements were made using ImageJ software (Wayne Rasband, NIH, USA) from photographs taken on an Olympus BX51 compound microscope mounted with DP25 camera attachment. Drawings were made with the aid of a drawing tube mounted on an Olympus compound microscope. For scanning electron microscopy (SEM) specimens were transferred to 2.5 % glutaraldehyde in 0.1 M phosphate buffer, then post-fixed in 1% osmium tetroxide and dehydrated through a gradient series of ethanols, critical-point dried in a CPD030 BalTec critical-point dryer (BalTec AG, Balzers, Liechtenstein) using carbon dioxide, mounted on aluminium stubs, and sputter coated with gold/palladium (60:40) to a thickness of 10 nm in an Emitech K575X Peltiercooled high-resolution sputter coater (EM Technologies, Ashford, Kent, UK). The specimens were viewed with a JEOL 6700 F field emission scanning electron microscope (JEOL Ltd., Tokyo, Japan) at the Otago Centre for Electron Microscopy (OCEM, University of Otago, New Zealand). Voucher specimens were deposited in Te Papa Museum, Wellington, New Zealand. All measurements are in micrometres throughout, and given as ranges with the mean in parentheses. ANOVAs were used to test for differences in various morphometrics of *Renicola* spp. between developmental stages, with Tukey post-hoc testing used to identify which pairs significantly differed between stages (XLSTAT, 2024 https://www.xlstat.com).

### Molecular data and analysis

To place the recovered *Renicola* specimens in a phylogenetic context, we sequenced DNA for *cox*1 and 28S genes of four individuals infecting little blue penguins, and two individuals infecting Fiordland crested penguins (Table 1). We also sequenced one representative of *Renicola* sp. metacercariae from a sprat *Sprattus muelleri* (Klunzinger), obtained from Oamaru Harbour in 2019. Genomic DNA was extracted using DNeasy® Blood and Tissue kit (Qiagen, Hilden, Germany) according to the manufacturer’s protocols. Primers trem.cox.rrnl (Králová-Hromadová et al., 2008) and T16 and T30 (Harper & Saunders, 2001), and JB3 (Bowles et al., 1993) were chosen for *cox*1 and 28S, respectively. Polymerase chain reaction (PCR) protocols for the *cox*1 gene consisted of 95 °C for 2–5 min, followed by 38–40 cycles of 95 °C for 30 sec–1 min, 48–50 °C for 30 sec–1 min and 72 °C for 1 min, and 72 °C for 10 min. Protocol for the 28S gene consisted of 94 °C for 5 min, 38 cycles of 94 °C for 30 sec, 45 °C for 30 sec and 72 °C for 2 min, and 72 °C for 7 min. PCR products were cleaned using EXOSAP^TM^ Express PCR Product Cleanup Reagent (USB Corporation, Cleveland, OH, USA) following the manufacturer’s instructions. Sanger sequencing by capillary electrophoresis was performed by the Genetic Analysis Service, Department of Anatomy, University of Otago (Dunedin, New Zealand).

**Table 1:** Data on *Renicola* spp. known from New Zealand, including new DNA sequences provided here, life stages, Isolate IDs and host species

Species	Isolate ID	Stage	Host species	28S	*cox*1
*Renicola websterae* **n. sp.**	FCP9tre1	Adult	Fiordland crested penguin	PV031508	PV031728
	LBP50tre1	Adult	Little blue penguin	PV031510	PV031729
	LBP45tre1A	Adult	Little blue penguin	PV031507	
	LBP45tre1B	Adult	Little blue penguin	PV031511	PV031727
	LBP56tre1	Adult	Little blue penguin	PV031512	PV031726
*Renicola* sp. 1 of Bennett et al. (2023)	FCP8tre1	Adult	Fiordland crested penguin	PV031509	PV031730
	ANC1tre1A_41	Metacercariae	Anchovy *Engraulis australis*	OQ407765	
	SPM8tre1A_41	Metacercariae	Sprat *Sprattus muelleri*	PV031506	
	SPM8tre1B_41	Metacercariae	Sprat *Sprattus muelleri*	PV031505	
*Renicola* sp. NZ of O’Dwyer et al. (2014)	AAPA2	Cercariae	Periwinkle *Austrolittorina antipodum*	KJ868215	KJ868205
*Renicola* sp. of Martorelli et al. (2008)		Cercariae	Snail *Zeacumantus subcarinatus*		

Sequences were imported to Geneious Prime ® v11.0.20.1, trimmed using the trim function with default parameters and manually edited for ambiguous bases. An alignment was created for the *cox*1 and 28S genes separately, together with close relatives downloaded from GenBank following BLASTn searches. Sequence GenBank identifiers are given on the illustrated phylogenies. The program jModelTest v2.1.6 (Guindon and Gascuel, 2003; Darriba et al., 2012) was used to estimate the model of evolution for each alignment, restricted to 3 substitution models compatible with MrBayes. For both alignments, GTR+I+G was selected as the best model. Bayesian inference was conducted in MrBayes version 3.2.7a (Huelsenbeck & Ronquist, 2001) using the online interface: Cyberinfrastructure for Phylogenetic Research (CIPRES) Science Gateway (Miller et al., 2010). Resulting trees were summarised in a 50% majority-rule consensus tree with clade credibility support values (Bayesian posterior probability, BPP) and branch length information. Trees were visualised in FigTree v1.4.4 (https://tree.bio.ed.uk//software/figtree/) and edited in Inkscape v1.3.2 (https://inkscape.org). BPP higher than 0.8 was considered moderately supported, and greater than 0.95 was considered strongly supported for nodal positioning. Uncorrected pairwise genetic distances were also estimated in MEGA v11 (Tamura et al., 2021).

## Results

### Developmental stages

**Fig.1:**
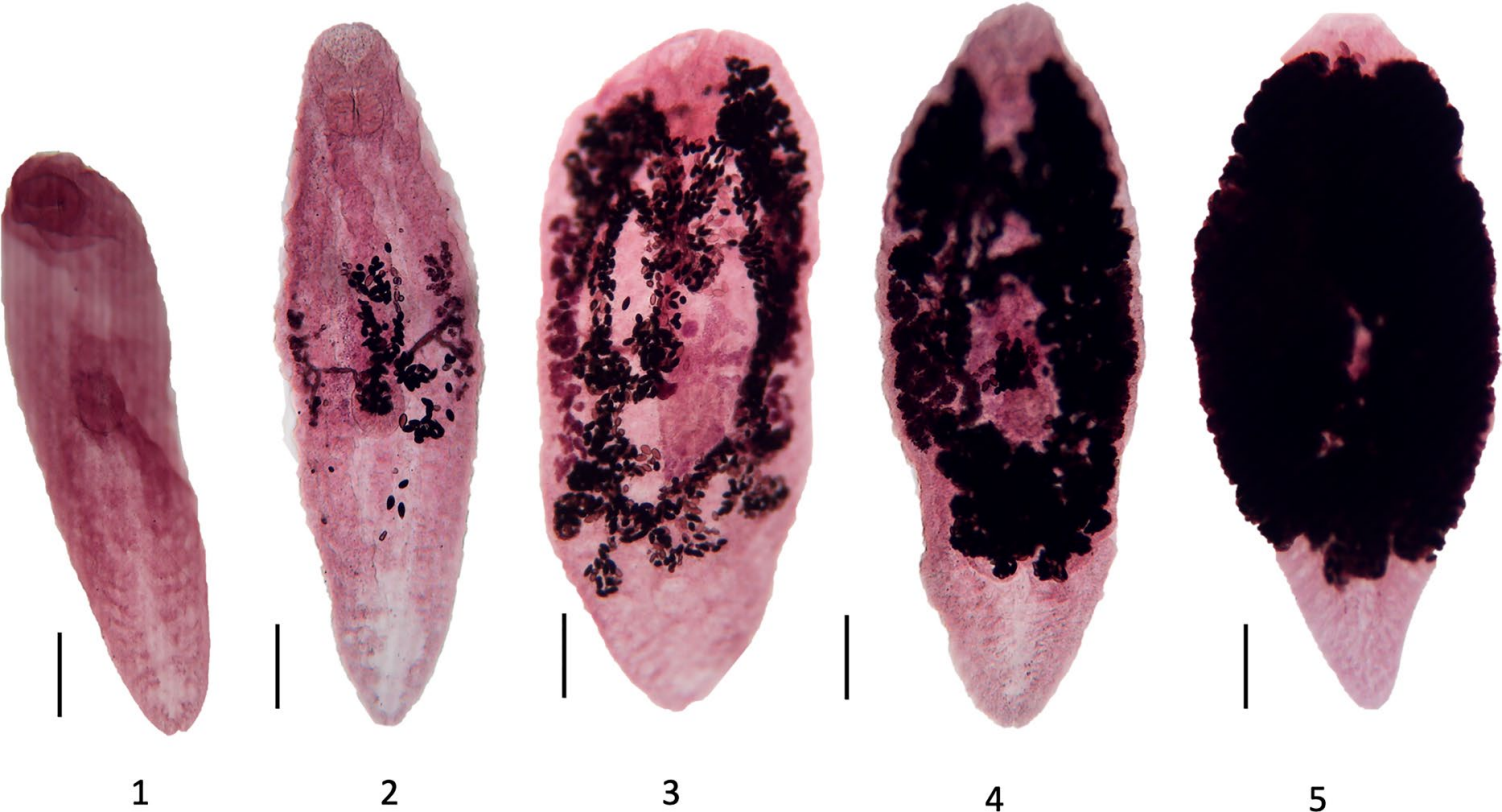
Specimens of *Renicola websterae* **n. sp.** artificially categorised into developmental ‘stages’; 1, 2, 3, 4 and 5. See text for explanation. Scale bars 200 µm.

As noted by other researchers (Dollfus, 1946; Gibson, 2008; Wright, 1956) fully mature adults of *Renicola* spp. are so heavily congested with eggs that details of the internal organs are obscured and usually impossible to observe. However, in earlier developmental stages when the uterus is only partly full of eggs, the organs are partly or wholly visible. By examining these earlier stages, we identified distinct developmental patterns that allowed us to categorise the specimens into artificial ‘stages’ (Fig. 1) as follows. Stage 1: newest infections show no sign of eggs or vitellaria, although genital primordia stain. Stage 2: vitellaria and vitelline ducts visible, although not necessarily in their entirety, a few eggs may be present in uterus. Stage 3: vitellaria reaching their maximum extent, with eggs distributed throughout most of uterus, organs still visible and assumed to be at or near their maximum size. Stage 4: uterus partially filled with eggs but with some areas unfilled and ventral sucker and organs remain partially visible. Stage 5: uterus filled with eggs, with only part of oral sucker and posterior quarter of body visible. ANOVA tests revealed that Stage 1 specimens differed significantly from Stages 3, 4, and 5 in body length (Tukey’s HSD results, p < 0.001). Additionally, Stage 1 specimens showed significant differences in sucker width ratios compared to Stages 2, 3, and 4 (Tukey’s HSD results, p < 0.05). No significant differences were found among the five stages for any other metrics tested; oral sucker width, ventral sucker width, pharynx width. As the principal characters did not differ significantly between stages 2–5, we combined measurements from these stages in the description, with the proviso that most organs were not visible in stage 5 specimens, and could not therefore be measured.

**Superfamily Microphalloidea Ward, 1901**

**Family Renicolidae Dollfus, 1939**

**Genus** ***Renicola*** **Cohn, 1904**

**Fig. 2:**
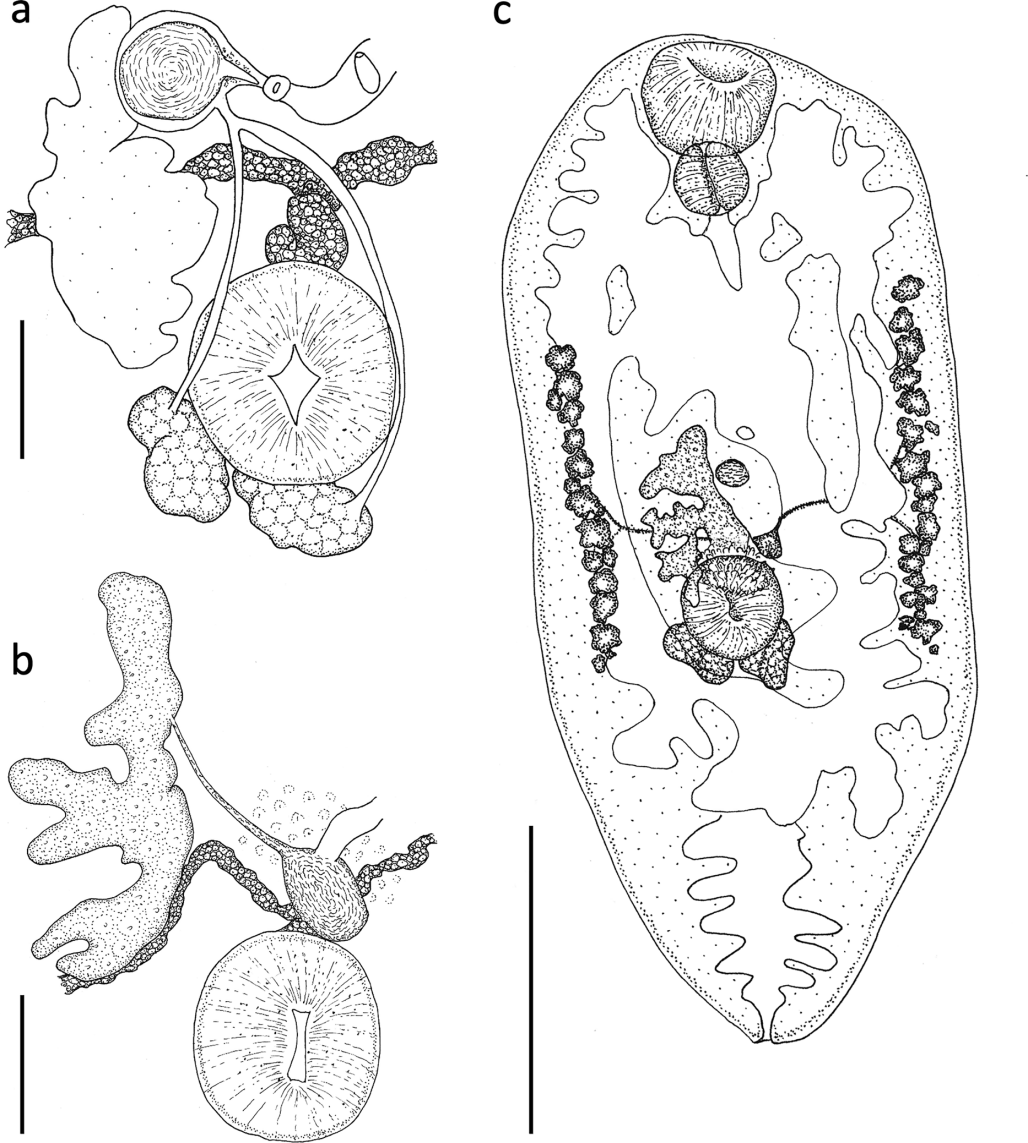
Drawings of *Renicola websterae* **n. sp.** **a** male reproductive system, **b** female reproductive system, **c** whole worm. Scale bars (**a**) and (**b**) 100 µm, (**c**) 500 µm

**Fig. 3:**
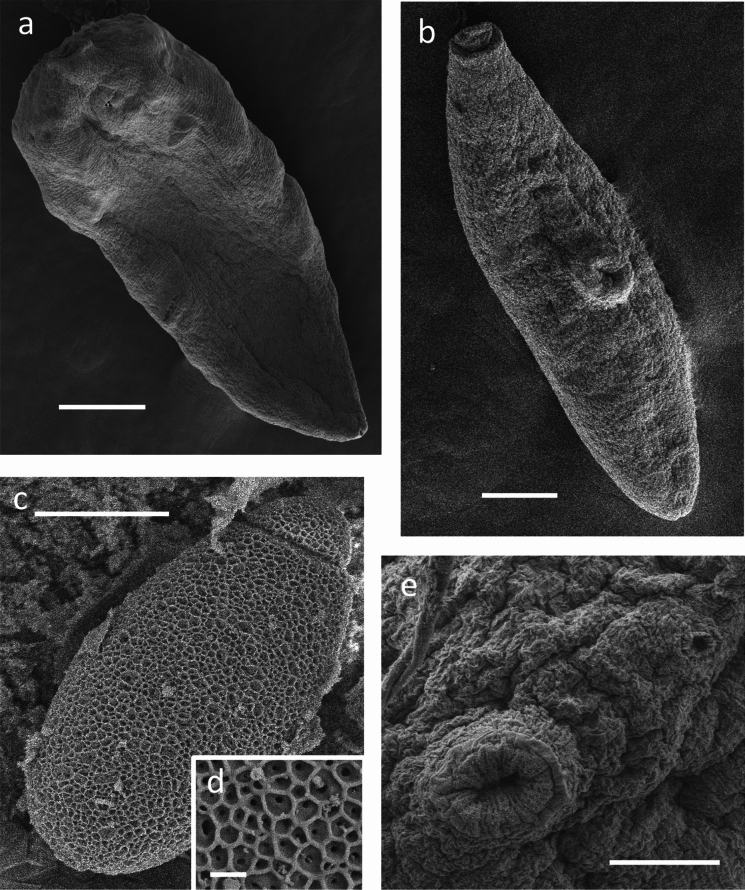
Scanning electron micrographs of *Renicola websterae* **n. sp.** **a** Dorsal view of mature specimen, **b** ventral view of immature specimen, **c** egg, **d** (insert) close-up of reticulated surface of egg with pores in the interstices, **e** close-up showing ventral sucker and genital pore.

***Renicola websterae* n. sp. (**Figs. 1–3**:** Table 2**)**

*Type host.* Otago little blue penguin *Eudyptula novaehollandiae* (Stephens) (Aves, Sphenisciformes, Spheniscidae)

*Other definitive hosts.* Fiordland crested penguin *Eudyptes pachyrhynchus* Gray

*Type locality.* Katiki Point, Otago, New Zealand 45.3919° S, 170.8671° E

*Other localities.* Long Beach 45.7560° S 170.6439° E, Tomahawk Beach 45.9060° S, 170.5446° E, Papanui Inlet 45.8456° S, 170.6981° E, Oamaru 45.1080° S, 170.9757° E, Otakou 45.8029 S, 170.7019° E, Waldronville 45.9250° S, 170.4084° E, Brighton 45.9481° S, 170.3333° E, Papatowai 46.5626° S, 169.4738° E (Otago), Waikawa Estuary 46.6270° S, 169.1503° E, Surat Bay 46.4773° S, 169.7310° E (Southland), Haast 43.8483° S, 169.0302° E (Westland).

*Site of infection.* Kidney tubules.

*Second intermediate host.* Unknown.

*First intermediate host.* Unknown.

*Prevalence.* 14 out of 25 little blue penguins (56%); 3 out of 10 Fiordland crested penguins (33.3%).

*Intensity.* 1–100+ (av. 56) in little blue penguins; 1–45 (av. 16) in Fiordland crested penguins.

*Type material*. Three slides comprising: 1) W.003976, Holotype, 2) W.003977, Paratypes (on same slide as Holotype), 3) W.003978, Paratypes, 4) W.003979, Paratypes.

*Representative DNA sequences.* See Table 1 for GenBank accession numbers of *cox*1 and 28S genes.

*Zoobank reference.* urn:lsid:zoobank.org:act:04D3080F-216F-47AA-8FBB-F5166E0D936F.

*Etymology.* The new species is named for Trudi Webster, until recently Conservation Science Adviser at the Yellow-eyed Penguin Trust, Otago, in recognition of her outstanding work, and that of the Trust, in their efforts in saving the endangered hoiho.

*Description* [based on 8 stage 2, 12 stage 3, 13 stage 4 and 16 stage 5 stained and mounted specimens]

Body oval, slipper-shaped or pyriform depending on developmental stage (see above and Fig. 1), maximum width at mid-body, dorsally concave often with inwardly curled margins, maximum thickness approximately one-eighth to one-quarter of body length, 1091–2240 (1754) long, 328–998 (651.5) wide; anterior three-quarters distended by caeca and egg-filled uterus in mature specimens; posterior quarter devoid of organs except excretory vesicle, rounded in immature specimens, more pointed in mature specimens, but not highly attenuated. Cuticle with no evidence of spines. Forebody (measured to centre of ventral sucker) 592–1219 (964.5) long. Hindbody 489–1025 (781.4) long. Posterior quarter without organs except excretory vesicle, rounded in immature specimens, more pointed in mature specimens, but not highly attenuated. Oral sucker subterminal, 140–217 (180.5) long by 139–288 (208.4) wide; opens directly into muscular, round pharynx; 91–133 (112.6) long by 91–133 (112.6) wide. Oesophagus short. Caeca extend to approximately halfway between posterior margin of ventral sucker and posterior margin of uterus. Ventral sucker small, muscular, 121–174 (148.5) long, 103–160 (137.8) wide; positioned at 50–60% of body length. Ratio of oral sucker width to ventral sucker width 1:1.16–1.92 (1:1.5). Testes 2, irregular to lobed, parallel or slightly oblique, close together posterior or postero-sinistral to ventral sucker; right 102–160 (122.3) long by 69–126 (92.7) wide; left 87–163 (127.1) long by 70–125 (91.7) (Fig. 2a). Vasa efferentia arise from anterior of testes and join to form vas deferens just prior to seminal vesicle. Seminal vesicle, large, round, stains strongly, surrounded by fine membrane, including prostatic gland cells, and ejaculatory duct, 40–92 (56.6) long and 39–90 (50.1) wide. Genital pore ventral, median or slightly sinistral, 119–205 (155.0) from anterior edge of ventral sucker (Fig. 3e. Ovary dextral, posterior end around middle of ventral sucker, rarely overlapping testes, 151–385 (251.4) long and 65–362 (162.3) wide; deeply lobed and irregular laterally, more so on distal border (Fig. 2b). Oviduct arising in middle third of ovary length. Seminal receptacle ventral to Mehlis’ gland. Laurer’s canal opening on dorsal surface in second quarter of body length. Uterus voluminous, making several ascending and descending passages, ventral to caeca, sometimes forms central sac, extends posteriorly to approximately 75–80% body length and anteriorly to level of oral sucker. Mature specimens filled to capacity with eggs rendering entire worm deeply coloured and obscuring all internal organs (Figs. 1 and 2c). Eggs operculated, very numerous, 28.7–36.9 (32.9) long by 16.5–19.8 (18.1) wide; opercular end narrower than non-opercular end. Surface of eggs with a dense reticulate network of ridges, forming lacunae each containing a single pore (Fig. 3c, d). Vitellaria extracaecal, mainly in middle third of body, extending from around anterior of ovary to around posterior of testes; small follicles of varying shapes and sizes, around 20–30, extent differing between right and left. Left vitellaria 320–699 (476.8) in length, right vitellaria 266–784 (447.7) in length. Vitelline duct traverses body just anterior to ventral sucker and has a central, prominent vitelline reservoir, and branches close to vitellaria. Excretory vesicle Y-shaped, extending from terminal excretory pore, dividing near posterior margin of uterus; bearing numerous lateral branches.

#### Remarks

Specimens of this new species, having been found in the kidneys of aquatic birds and consistent with the diagnosis as given by Gibson (2008) were identified as belonging to genus *Renicola*. The specimens occurred in pairs, in cyst-like capsules within the kidney tubules, which sometimes also contained many released eggs.

Tegumental spines are included as a diagnostic character for *Renicola* (see Gibson, 2008). Nevertheless, some species are described as having no spines, and in others this feature is not mentioned or illustrated (Wright, 1956). Despite thorough examination of fresh, mounted and scanning electron microscope specimens, we found no evidence of tegumental spines or associated scars (Fig. 3). Given that spines may often be lost upon fixation, the presence or absence of this feature in the literature may be unreliable. Therefore, we have opted not to use tegumental spines as a comparative character in our analysis.

As noted by Galaktionov et al. (2023) and Heneberg et al. (2016), species within the ‘sloanei’ group exhibit high morphological similarity, suggesting the presence of multiple species-complexes. Specifically, Galaktionov et al. (2023) highlighted the broad host range found for *R*. *keimahuri* Yamaguti, 1939 leading to the suggestion that this species may comprise cryptic species, whereas Heneberg et al. (2016) referred to the group comprising *R*. *lari* Timon-David, 1933, *R*. *pinguis* (Mehlis, 1846), *R*. *sternae* Sitko & Heneberg, 2016, and *R*. *sloanei* Wright, 1954, which, although genetically distinct, are highly similar in morphology.

Although many metrics overlap among the* ‘*sloanei’ species (Table 2) there are distinguishable points of difference*. Renicola websterae* is distinct from other species in the ‘sloanei’ group in its ventral-to-oral sucker width ratio [1:1.21.9 (1.5)]. The ventral sucker is significantly larger than that in *R*. *keimahuri*, *R*. *lari*, *R*. *pinguis*, and *R*. *sternae*, which have ventral sucker width ratios of 1:2.0–3.3, 1:1.6–4.0 (2.8), 1:2.0–5.6 (3.9), and 1:2.0–4.0 (2.5), respectively. In *R*. *sloanei*, although the ventral sucker width overlaps with that of *R*. *websterae*, the oral sucker is much larger, resulting in a ratio of 1:2.2–2.5. Furthermore, the egg sizes of *R*. *keimahuri* (39–45 × 20–22 µm), *R*. *lari* (48–50 × 27–28 µm), and *R*. *pinguis* (51–55 × 23–26 µm) are significantly larger compared to those of *R*. *websterae*. All of the species in the ‘sloanei’ complex have a highly attenuated ‘caudal extension’ which tapers to a sharpish point. Only fully mature specimens of *R*. *websterae* could be said to have a caudal extension, which is not as sharply pointed as found in other species.

**Table 2:** Measurements of *Renicola* species from the ‘sloanei’ group of species (Clade II of phylogenies). Measures are in µm except for body length

Species	*Renicola websterae* **n. sp.** [stages 2–5]	*Renicola* sp. 1	*Renicola caudescens*	*Renicola foliata*	*Renicola keimahuri*	*Renicola lari*^*a*^	*Renicola pinguis*^*a*^	*Renicola sloanei*	*Renicola sternae*
Author	This study	This study	Ching, 1989	Ching, 1989	Yamaguti, 1939	Timon-David, 1933	(Mehlis, 1846) Cohn, 1904	Wright (1954)	Sitko & Heneberg, 2016
Host	little blue and Fiordland crested penguins	Fiordland crested penguin	Noddy	Shearwater, noddy	Seabirds	Gulls, terns, mergus	Crested grebe	Penguins	Terns
Locality	NZ	NZ	Australia	Australia	Japan & far East Russia	N & W Europe, Russia	N & W Europe	Southern Hemisphere	Czech Rep.
BL (mm)	1.1–2.2 (1.75)	2.47	0.5–1.5 (0.96)	1.2–2.2 (1.77)	1.1–2.1	1.0–1.8 (1.3)	1.28–2.57 (2.1)	1.47–2.7	0.57–1.63 (1.1)
WidestW	328–998 (651.5)	640	303–857 (560)	1.0–1.3 (1.13)	470–1000	522–1000 (794)	857–1714 (1200	690–1260	514–1057 (785)
FBL	592–1219 (964.5)	1363		591–1000 (875)		551–1118 (767)	857–1571 (1212		422–1143 (741)
HBL	489–1025 (781.4)	1106		632–1183 (894).		322–745 (484)	714–1143 (762		232–552 (367)
FBL/HBL	0.98–1.72 (1.25)	1.23				1:1·1–2·0 (1·6)	1:1·0–1·8 (1·4		1:1·1–2·9 (2·0)
OSL	140–217 (180.5)	205	82–155 (106)	139–237 (167)		174–296 (220)	200–398 (309	257–329	145–285 (198)
OSW	139–288 (208.4)	245	115–237 (145)	188–270 (233)	150–200d	203–348 (266)	230–538 (419	229–286	174–368 (242)
PhL	91–133 (112.6)	104	33–94 (68)	65–123 (101)		70–104 (89)	87–161 (123)	114	48–87 (67)
PhW	97–148 (121.7)	132	56–123 (79)	90–139 (123)	40–60	70–99 (79)	70–161 (113	114	36–87 (65)
VSL	121–174 (148.5)	165	56–123 (79)	106–164 (128)		81–145 (98)	70–140 (106		84–108 (96)
VSW	103–160 (137.8)	158	33–140 (67)	98–147 (133)	60–70	81–145 (98)	70–129 (109	114–129	84–110 (96)
OS:VS	1.16–1.92 (1.5)	1.55	0.46–0.74	1:0.5–0.6	0.3–0.5	1:1·6–4·0 (2·8)	1:2·0–5·6 (3·9		0.25–0.5 (0.4)
LvitL	320–699 (476.8)	–			340–500	232–598 (356)	368–743 (530		261–506 (352)
RvitL	266–784 (447.7)	–			220	249–539 (355)	457–745 (575		261–506 (352)
TestRL	102–160 (122.3)	–	51–107 (65)	49–112 (79)	110–120	70–122 (100)	87–269 (177		80–116 (95)
TestRW	69–126 (92.7)	–	47–65 (50)	41–82 (64)	90	48–70 (59)	58–215 (130		58–74 (66)
TestLL	87–163 (127.1)	–		41–102 (86)		70–122 (100)	87–297 (181		80–116 (97)
TestLW	70–125 (91.7)	–		33–71 (54)		48–70 (59)	58–260 (130		58–74 (66)
OvL	151–385 (251.4)	–	74–211 (139)	139–237 (164)	260–290	167–290 (223)	145–430 (271		87–261 (199)
OvW	65–362 (162.3)	–	41–153 (102)	82–172 (103)	110–130	104–203 (135)	116–269 (188		87–145 (112)
Eggl	28.7–36.9 (32.9)	–	20–31 (26)	26–36 (31.6)	39–45	46–48 (47)	51–55 (53	28–34	32–36 (35)
EggW	16.5–19.8 (18.1)	–	13–18 (14)	15–20 (16.7)	20–22	26–28 (27)	23–26 (24	16–18	17–24 (22)

^a^Data from Heneberg et al. (2016)

*Renicola websterae* is closest to *R*. *sloanei* in terms of shape, measurements, genetic distance, and its use of penguin hosts. In addition to the sucker ratio difference, *R*. *sloanei* exhibits caeca that extend almost to the posterior extremity, whereas the caeca in *R*. *websterae* extend only to slightly below the ventral sucker. Furthermore, the extent of the vitellaria is greater in *R*. *sloanei*, reaching anteriorly almost to level with the pharynx and terminating just above the posterior extent of the uterus, whereas in *R*. *websterae* the vitellaria are far shorter. Furthermore, *R. sloanei* was found in penguins from South Africa and Manx shearwater *Puffinus puffinus* (Brünnich) in Brazil, whereas *R*. *websterae* is, so far, known to occur only in New Zealand.

Two named species of *Renicola* have been reported from Australia, the nearest landmass to New Zealand, and the locality most likely to harbour comparable species of parasites (Ching, 1989). Both were found infecting the blue noddy *Anous minutus* Boie. *Renicola caudescens* Ching, 1989 is smaller in all metrics than *R*. *websterae*, except for width, with the oral sucker width 115–237 (145) and ventral sucker width 33–140 (67) showing no overlap in measurements with those of *R*. *websterae.* Additionally, the form and position of the vitellaria (6 large follicles, overlapping caeca) distinguish *R*. *caudescens* from the specimens described herein. The second Australian species, *R*. *foliata* Ching, 1989, is notably rounded and leaf-shaped, with a width ranging from 1.0 to 1.3 mm (mean 1.13 mm), significantly larger than that of *R*. *websterae*, despite having a similar overall body length. Currently, neither of the Australian species is represented by genetic sequences for molecular comparison.

### Molecular remarks

**Fig. 4:**
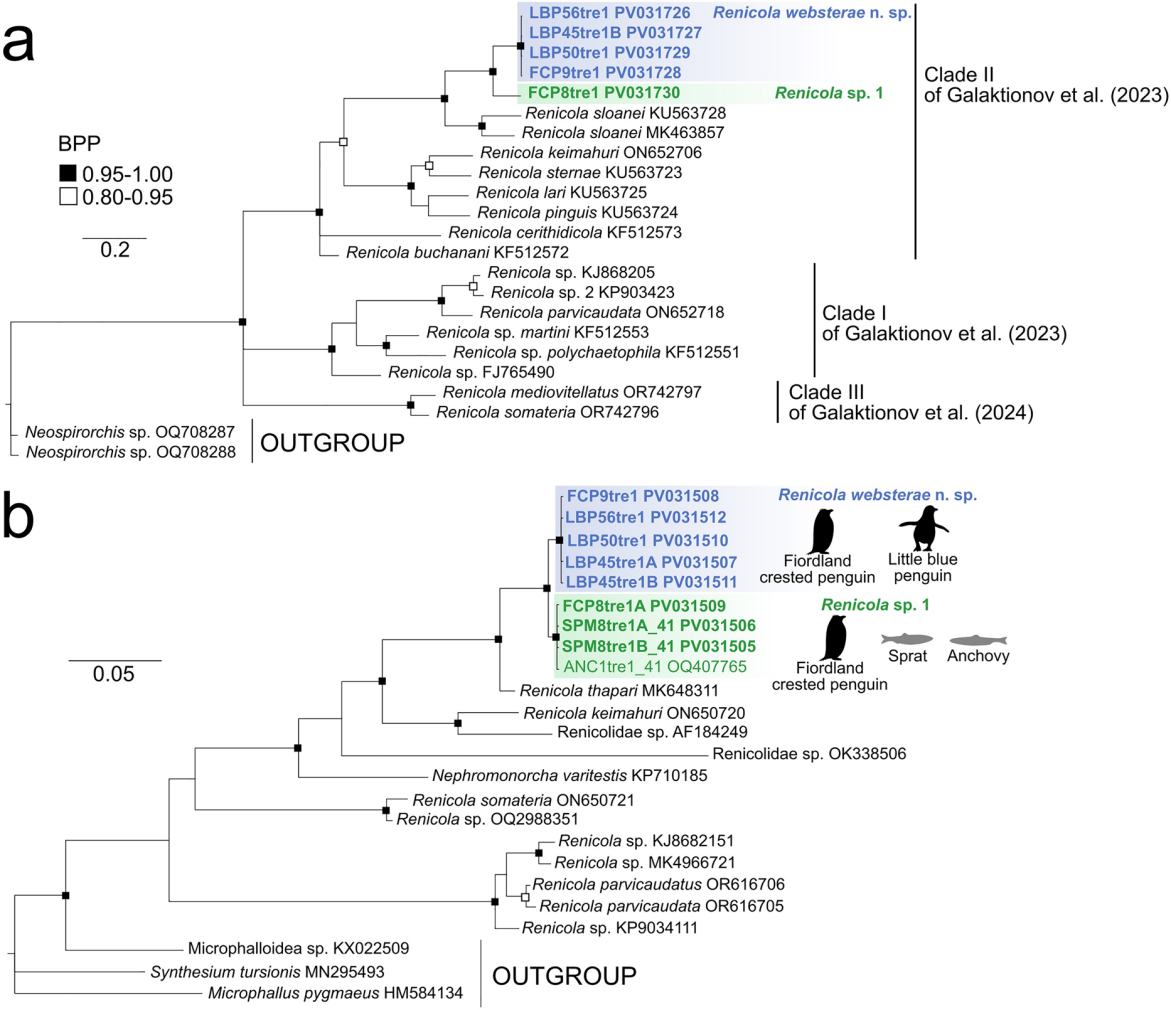
50% majority rule Bayesian inference phylogenetic trees inferred from **a** *cox*1 gene data and **b** 28s data of species within *Renicola* genus.

Galaktionov et al. (2023) generated a *cox1* phylogeny that divided the *Renicola* species into two major clades. Clade II (their ‘sloanei’ clade) comprised *R*. *sloanei*, *R*. *pinguis*, *R*. *sternae*, *R*. *lari*, and two species known only from their cercarial stages, *R*. *buchanani* and *R*. *cerithidicola* Martin, 1971. In our *cox*1 phylogeny, *R*. *websterae* was found within Galaktionov et al.’s Clade II, forming a subclade with *R*. *sloanei* with high nodal support (Fig. 4a). A sequence from *R*. *keimahuri* from a gull in Russia (ON652706) was also placed in Clade II. The mean genetic divergence between *R*. *sloanei* sequences was 10.2%, and the mean distance between *R*. *sloanei* and *R*. *websterae* was 16.1%. Within *cox*1, we observed no genetic variation in the four samples amplified. Between all species of *Renicola* for which DNA sequences are available, the mean genetic distance between species ranged from 7.82 to 37.72%, with an average of 23.7% between species. A more recent phylogeny (Galaktionov et al. 2024) included sequences from *R*. *somateria* and *R*. *mediovitellata*, renicolids of ducks, which formed a third clade, as in Fig. 4a.

Our 28S phylogeny showed high support for *Renicola websterae* as a sister taxon to the *Renicola* sp. infecting Fiordland crested penguins from this study (*Renicola* sp. 1 of Bennett et al. (2023), and both formed a sister clade to *R*. *thapari* Caballero, 1953 from a booby in Mexico (Fig. 4b). Mean genetic divergence estimates between species within *Renicola* had a large range from 1.35–54.56% across the two Clades defined by Galaktionov et al. (2023). Within Clade II, *Renicola websterae* exhibited 5.04% genetic divergence with *R*. *thapari.*

#### ***Renicola*** sp. 1 of Bennett et al. (2023)

A second, phylogenetically distinct species (Fig. 4) was discovered as an adult from a Fiordland crested penguin and a sprat (as metacercariae), sequences from which were identical to those from metacercariae from an anchovy, previously genetically characterised in Bennett et al. (2023). One single specimen from the same host penguin was assumed to represent this genotype. Measurements from this fresh, unstained, specimen are given in Table 2. Although it is immature (with no visible gonads, vitellaria, or eggs), it is longer than the longest immature (Stage 1) specimens of *R*. *websterae*, and the sucker sizes are at the upper end of the size range for *R*. *websterae*. More specimens are required to fully evaluate this species. Based on DNA sequencing, the *cox*1 and 28S phylogenies place this species as a sister taxon to *R*. *websterae* with high nodal support. If this species is, indeed, distinct from *R*. *websterae* then the two species are genetically closer to each other than are other *Renicola* species, with 1.35% divergence in *cox*1 and 8.34% divergence in 28S data.

## Discussion

Renicolids have a simple basic structure, leaving little room for interspecific character variation. Furthermore, what distinguishing characters do exist are often obscured by the gravid uterus and are subject to state of maturity, and whatever preservation techniques are employed (Wright, 1956). However, our examination of non-fully gravid specimens has revealed consistency among our samples, allowing for meaningful morphological comparisons with other species. Although we do not wish promote the practice of using immature specimens for the erection of new species generally, we propose that, in this taxon, sub-mature specimens can be effectively utilised for taxonomic measurements without significantly skewing the data. As species of *Renicola* are notoriously difficult to describe when fully mature, we hope that our artificial categorisation of developmental stages may assist future renicolid workers when describing new species.

This study provides a morphological description of a new species of *Renicola*, and a molecular description of both *Reniciola websterae* and another undescribed *Renicola* sp. 1 of Bennett et al. (2023). Although *Renicola websterae* is commonly found in Fiordland crested and little blue penguins we are yet to identify the molluscan first, or teleost second intermediate hosts for this species. However, we match two life stages (adult and metacercariae) of the second, undescribed, species using molecular data, identifying a probable predator prey interaction between the fishes anchovy (*Engraulis australis*) and sprat (*Sprattus muelleri*), and Fiordland crested penguins (Bennett et al., 2023).

Our phylogenies of 28S and *cox*1 genes are congruent with those found by Galaktionov et al. (2023) and support the relationships among the Clade II (‘sloanei’) species. The species included are those comprising the “pinguis” group of Odening (1962) which was also supported by Heneberg et al. (2016). Other, unnamed, renicolids from New Zealand have been sequenced from cercarial stages in previous studies (Martorelli et al., 2008; O’Dwyer et al., 2014), which are phylogenetically distinct from the species described here, being placed in Galaktionov et al.’s (2023) Clade 1. We have yet to discover the adults of these species.

Our 28S phylogenetic tree indicates that *Renicola* is paraphyletic in relation to *Nephromonorcha*, supporting the findings of Galaktionov et al. (2023). The genus *Nephromonorcha* is characterised solely by the presence of a single testis; however, there is considerable evidence that several species within this genus include individuals with contiguous testes. This variability, along with the challenges in fully observing the testes, makes this characteristic questionable for distinguishing between the two genera in this case. Gibson (2008) only tentatively recognised *Nephromonorcha* as a valid genus, and Galaktionov et al. (2023) maintained its generic distinction despite acknowledging the variability in testes merging among some species, and illustrating its paraphyletic status in their phylogenetic analysis. As we have not examined specimens of *Nephromonorcha* for this study, we refrain from synonymising the genera at this time. However, we recommend that the morphology of the current species of *Nephromonorcha* be re-evaluated to confirm its congenericity with *Renicola*.

We have dissected various penguin species over the past few years for their parasitic fauna (manuscript in prep), including four erect crested penguins *Eudyptes sclateri* Buller, three Snares crested penguins *Eudyptes robustus* Oliver, and thirty yellow-eyed penguins *Megadyptes antipodes* (Hombron & Jacquinot). However, *Renicola websterae* was found only in little blue and Fiordland crested penguins. Why are liver flukes absent in other penguins and rare in Fiordland penguins? It is possible that renicolid species are strongly host-specific, and that stronger immune responses or lower susceptibility of the hosts prevent the flukes from establishing in the kidneys of other penguins. Although most *Renicola* species in the literature are documented from a single host, suggesting they exhibit relatively high specificity, some have been reported from multiple hosts. However, evaluating records of multiple-host species is complicated, as these cases may well involve cryptic *Renicola* species that require further examination. For example, *R*. *keimahuri* has been reported from a wide variety of marine birds (see Sudarikov & Stenko, 1984), presumably identified as such morphologically, but without the benefit of DNA sequence to identify molecular differences. A broader range of samples within the genus is needed to ascertain exactly how much molecular variability exists within and between other species. We suspect, given the relatively large genetic distance within and between species at the level of both 28S and *cox*1 markers, a greater diversity of species is awaiting discovery among those reported, and speculate that strong host-specificity is the norm for this family.

Alternatively, the absence of renicolid infections in other New Zealand penguins could be related to their feeding habits. The diet of Fiordland crested and little blue penguins (at least from Southern New Zealand) overlaps by 46%, although their overlap with yellow-eyed penguins is only 18% and 17%, respectively (Van Heezik, 1990). Additionally, yellow-eyed penguins tend to consume fewer large prey items, whereas Fiordland crested and little blue penguins eat a greater number of smaller items. These dietary differences could explain the absence of renicolids in other penguin species. Such trends raise concerns about future infections with changing prey-availability, especially for yellow-eyed penguins whose dietary composition has had marked changes over the last few years, coinciding with dramatic population declines (Boessenkool et al., 2010). Although yellow-eyed penguins were not infected with *R*. *websterae,* forced changes in diet due to climate change could make them vulnerable to infection, which could potentially prove pathogenic.

Crockett and Kearns (1975) reported heavy burdens of flukes in the kidneys of little blue penguins wrecked in large numbers in Northland, New Zealand. They found that, although there was minimal apparent pathology, the parasites exacerbated the weakness of the birds, thus played a role in their mortality. It is likely that this report referred to one of the *Renicola* species described herein. In other bird hosts, renicolids have been associated with necrosis, or renal inflammation (Mahdy & Shaheed, 2001; Jerdy et al., 2016). Together, this raises further questions about the pathogencity of renicolids in New Zealand penguins, making it important that knowledge of their existence is passed on to wildlife veterinarians and agencies.

## Data Availability

DNA sequences have been accessioned to GenBank, under accessions PV031505–512 and PV031726–730. Voucher specimens have been accessioned at Te Papa Museum; accession numbers W.003976–79.
